# (Tetra­oxidoselenato-κ*O*)tris­(thio­urea-κ*S*)zinc(II)

**DOI:** 10.1107/S1600536808000743

**Published:** 2008-01-11

**Authors:** Radmila Krupková, Jan Fábry, Ivana Císařová, Přemysl Vaněk

**Affiliations:** a Institute of Physics, Academy of Sciences of the Czech Republic, Na Slovance 2, 182 21 Praha 8, Czech Republic; b Department of Inorganic Chemistry, Faculty of Science, Charles University, Hlavova 8, 128 43 Praha 2, Czech Republic

## Abstract

The title structure, [Zn(SeO_4_)(CH_4_N_2_S)_3_], is isomorphous with sulfatotris(thio­urea)zinc(II). In both structures, the Zn^2+^ cation is coordinated in a tetra­hedral geometry. The corresponding intra­molecular distances are quite similar except for the Se—O and S—O distances. Although the hydrogen-bonding patterns are similar, there are some differences; in the title structure all the H atoms are involved in the hydrogen-bond pattern, in contrast to the situation in sulfatotris(thio­urea)zinc(II). No reproducible anomalies were detected by differential scanning calorimetry in the range 93–463 K until decomposition started at the higher temperature.

## Related literature

For related literature, see: Krupková *et al.* (2007 ▶); Alex & Phillip (2001 ▶); Becker & Coppens (1974 ▶); PerkinElmer (2001 ▶); Ramabadran *et al.* (1992 ▶); Ushasree *et al.* (1998 ▶, 2000 ▶); Venkataramanan *et al.* (1995 ▶).
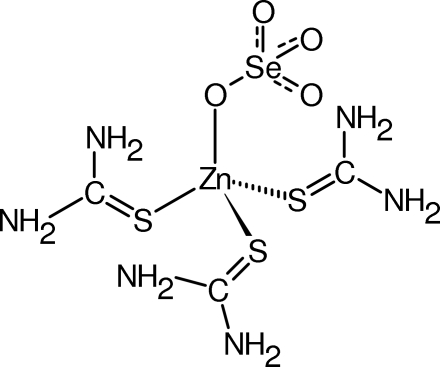


## Experimental

### 

#### Crystal data

[Zn(SeO_4_)(CH_4_N_2_S)_3_]
*M* *_r_* = 436.7
Orthorhombic, 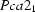

*a* = 11.2045 (2) Å
*b* = 7.8824 (1) Å
*c* = 15.7960 (2) Å
*V* = 1395.08 (4) Å^3^
*Z* = 4
Mo *K*α radiation
μ = 4.83 mm^−1^
*T* = 292 K
0.35 × 0.25 × 0.1 mm

#### Data collection

Nonius KappaCCD diffractometer
Absorption correction: Gaussian (Coppens & Hamilton, 1970 ▶) *T*_min_ = 0.223, *T*_max_ = 0.602
23449 measured reflections
3150 independent reflections
3004 reflections with *I* > 3σ(*I*)
*R*_int_ = 0.038

#### Refinement

*R*[*F* ^2^ > 2σ(*F* ^2^)] = 0.022
*wR*(*F* ^2^) = 0.050
*S* = 1.52
3150 reflections
163 parameters
H-atom parameters constrained
Δρ_max_ = 0.36 e Å^−3^
Δρ_min_ = −0.25 e Å^−3^
Absolute structure: Flack (1983 ▶), 1492 Friedel pairs
Flack parameter: −0.020 (6)

### 

Data collection: *COLLECT* (Hooft, 1998 ▶); cell refinement: *SCALEPACK* (Otwinowski & Minor, 1997 ▶); data reduction: *SCALEPACK* and *DENZO* (Otwinowski & Minor, 1997 ▶); program(s) used to solve structure: *SIR97* (Altomare *et al.*, 1997 ▶); program(s) used to refine structure: (*JANA2000*; Petříček *et al.*, 2000 ▶); molecular graphics: *PLATON* (Spek, 2003 ▶); software used to prepare material for publication: *JANA2000*.

## Supplementary Material

Crystal structure: contains datablocks global, I. DOI: 10.1107/S1600536808000743/bq2062sup1.cif

Structure factors: contains datablocks I. DOI: 10.1107/S1600536808000743/bq2062Isup2.hkl

Additional supplementary materials:  crystallographic information; 3D view; checkCIF report

## Figures and Tables

**Table 1:** Hydrogen-bond geometry (Å, °)

*D*—H⋯*A*	*D*—H	H⋯*A*	*D*⋯*A*	*D*—H⋯*A*
N1—H1N1⋯O4^i^*	0.89	2.20	3.066 (3)	164
N1—H2N1⋯O3^ii^*	0.89	2.38	3.072 (3)	135
N2—H1N2⋯O2^ii^*	0.89	1.98	2.852 (3)	167
N2—H2N2⋯S3	0.89	2.63	3.497 (3)	166
N3—H1N3⋯O3*	0.89	2.17	2.988 (3)	152
N3—H2N3⋯O1^iii^*	0.89	2.04	2.895 (3)	160
N4—H1N4⋯O2^iv^*	0.89	2.12	2.999 (3)	168
N4—H2N4⋯S2^iii^	0.89	2.86	3.643 (2)	148
N5—H1N5⋯O3^v^*	0.89	2.06	2.938 (3)	170
N5—H2N5⋯O4^vi^	0.89	2.57	3.297 (3)	139
N6—H1N6⋯S1	0.89	2.73	3.577 (3)	159
N6—H2N6⋯O4^v^*	0.89	2.19	2.905 (3)	137

Symmetry codes: (i) 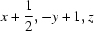; (ii) 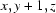; (iii) 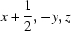; (iv) 
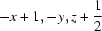
; (v) 
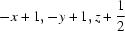
; (vi) 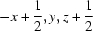. * indicates that the pertinent hydrogen bond is also present in Zn[(SC(NH_2_)_2_]_3_(SO_4_) (Krupková *et al.*, 2007 ▶).

**Fig. 1.**
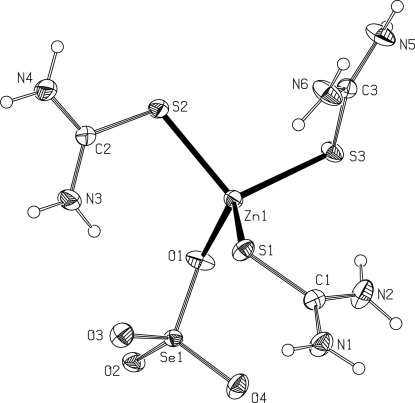
View of the title molecule with anisotropic displacement parameters shown at the 30% probability level.

## supplementary crystallographic information

### Comment

Zinc [tris(thiourea)]sulfate, isomorphous to the title structure, is reported to be a perspective semiorganic non-linear optical material (Ushasree *et al.*, 1998, 2000). It can substitute potassium dihydrogenphosphate in technical applications (Ramabadran *et al.*, 1992; Alex & Phillip, 2001). It has also an exceptionally wide acceptance angle for second harmonic generation (Ramabadran *et al.*, 1992). Its resistance against laser induced damage is good (Venkataramanan *et al.*, 1995).

We have synthesized the title compound since it is expected that it might show similar interesting properties as its known isostructural counterpart (Krupková *et al.*, 2007). As a part of our on-going study of the title compound we report here its structure determination. The investigation od dielectric and optical properties is in progress.

The common features and differences between the hydrogen-bond patterns in both isostructural compounds are shown in Tab. 1. This table shows that the stronger hydrogen bonds are common for both isostructural compounds.

### Experimental

The title compound has been prepared in a similar way as zinc[tris(thiourea)] sulfate. The preparation was carried out in two steps according to following equations:

(1) [ZnCO_3_][Zn(OH)_2_] + 2H_2_SeO_4_ + 3H_2_O → 2ZnSeO_4_.6H_2_O + CO_2_

(2) 4ZnSeO_4_.6H_2_O + 3 CS(NH_2_)_2_→ Zn[CS(NH_2_)_2_]_3_}[SeO_4_]

5.0 g (0.222 *M*) of ZnCO_3_[Zn(OH)_2_] dissolved in 3.6 g (0.2 *M*) of distilled H_2_O reacted with 6.45 g (96%) H_2_SeO_4_ (0.0427 *M*) at room temperature. After the neutralization white suspension was obtained. The suspension into which had been poured 50 ml of distilled H_2_O was heated at 60°C for 30 minutes. The solution became clearer and its pH=4.

Then, at 50°C was added 10.14 g (0.1332 *M*) of thiourea. The solution became orange-coloured and under stirring it was kept at 50°C for another 10 minutes. An orange precipitate has developed to which another 100 ml of distilled water was added. The mixture was stirred for another 20 minutes and then cooled down to room temperature. After two days, transparent crystals of length of 0.5 mm appeared at the walls of the beaker while an orange precipitate covered its bottom. Next day the precipitate was filtered off, some orange-tinged crystals have been isolated as seeds that were introduced into the filtrate. After a week clear transparent crystals appeared of the size of 1 cm, of the similar *HABITUS* as zinc[tris(thiourea)] sulfate (Alex & Phillip, 2001).

### Refinement

All the H atoms were discernible in the difference Fourier map and even could be refined. Nevertheless, their coordinates were constrained in riding motion formalism: The pertinent distances equalled to 0.89Å and *U*_iso_(H)=1.2*U*_eq_(N).

The calorimetric experiments were performed on PerkinElmer DSC 7 and Pyris Diamond differential scanning calorimeters using PYRIS Software (PerkinElmer, 2001), with m = 30 mg, a temperature interval of 93–466 K and scanning rate of 10 K/min. No reproducible DSC anomalies were detected until the symptoms of decomposition at 463 K.

### Crystal data

**Table:**

[Zn(SeO_4_)(CH_4_N_2_S)_3_]	*F*_000_ = 864
*M**_r_* = 436.7	*D*_x_ = 2.079 Mg m^−^^3^
Orthorhombic, *P**c**a*2_1_	Mo *K*α radiation λ = 0.71069 Å
Hall symbol: P 2c -2ac	Cell parameters from 14388 reflections
*a* = 11.2045 (2) Å	θ = 1.0–27.5º
*b* = 7.8824 (1) Å	µ = 4.83 mm^−^^1^
*c* = 15.7960 (2) Å	*T* = 292 K
*V* = 1395.08 (4) Å^3^	Prism, colourless
*Z* = 4	0.35 × 0.25 × 0.1 mm

### Data collection

**Table:**

Nonius KappaCCD diffractometer	3150 independent reflections
Radiation source: fine-focus sealed tube	3004 reflections with *I* > 3σ(*I*)
Monochromator: graphite	*R*_int_ = 0.039
*T* = 292 K	θ_max_ = 27.5º
ω scans	θ_min_ = 3.2º
Absorption correction: Gaussian(Coppens & Hamilton, 1970)	*h* = −14→14
*T*_min_ = 0.223, *T*_max_ = 0.602	*k* = −10→10
23449 measured reflections	*l* = −19→20

### Refinement

**Table:**

Refinement on *F*^2^	Weighting scheme based on measured s.u.'s *w* = 1/[σ^2^(*I*) + 0.0004*I*^2^]
*R*[*F*^2^ > 2σ(*F*^2^)] = 0.022	(Δ/σ)_max_ = 0.005
*wR*(*F*^2^) = 0.050	Δρ_max_ = 0.36 e Å^−^^3^
*S* = 1.52	Δρ_min_ = −0.25 e Å^−^^3^
3150 reflections	Extinction correction: B-C type 1 Lorentzian isotropic (Becker & Coppens, 1974)
163 parameters	Extinction coefficient: 1.65 (5)
48 constraints	Absolute structure: Flack (1983), 1492 Friedel pairs
H-atom parameters constrained	Flack parameter: −0.020 (6)

### Special details

**Table:**

Refinement. The Flack parameter converged to the value -0.020 (6), so it was excluded from the final refinement.

### Fractional atomic coordinates and isotropic or equivalent isotropic displacement parameters (Å^2^)

**Table:**

	*x*	*y*	*z*	*U*_iso_*/*U*_eq_	
Zn1	0.43588 (3)	0.34032 (3)	0.42203 (2)	0.02479 (9)	
Se1	0.37373 (2)	0.15124 (3)	0.247798	0.02293 (7)	
O1	0.34191 (19)	0.1846 (2)	0.34926 (13)	0.0363 (6)	
O2	0.29853 (18)	−0.0173 (2)	0.22066 (12)	0.0317 (6)	
O3	0.51627 (17)	0.1169 (2)	0.23987 (16)	0.0372 (6)	
O4	0.33169 (19)	0.3147 (2)	0.19315 (15)	0.0392 (6)	
S1	0.57849 (6)	0.49589 (8)	0.34906 (5)	0.03060 (19)	
C1	0.5054 (2)	0.6544 (2)	0.29483 (16)	0.0290 (8)	
N1	0.5667 (2)	0.7339 (3)	0.23498 (16)	0.0401 (8)	
N2	0.3957 (2)	0.7021 (3)	0.31154 (18)	0.0447 (9)	
S2	0.52313 (7)	0.17226 (9)	0.52667 (5)	0.0327 (2)	
C2	0.64030 (18)	0.0636 (3)	0.48127 (17)	0.0314 (8)	
N3	0.6501 (2)	0.0405 (4)	0.39895 (16)	0.0468 (9)	
N4	0.7222 (2)	0.0025 (3)	0.53155 (17)	0.0460 (9)	
S3	0.29592 (6)	0.50137 (8)	0.49583 (5)	0.03039 (18)	
C3	0.3845 (2)	0.5992 (3)	0.57094 (15)	0.0295 (7)	
N5	0.3360 (3)	0.6436 (3)	0.64329 (16)	0.0453 (9)	
N6	0.4979 (2)	0.6351 (3)	0.55684 (18)	0.0455 (9)	
H1n1	0.63954	0.698311	0.22123	0.0482*	
H2n1	0.534956	0.822806	0.208546	0.0482*	
H1n2	0.359482	0.77757	0.278428	0.0536*	
H2n2	0.35742	0.658874	0.355973	0.0536*	
H1n3	0.59099	0.071809	0.364689	0.0562*	
H2n3	0.71576	−0.006424	0.377612	0.0562*	
H1n4	0.718708	0.022995	0.58691	0.0552*	
H2n4	0.781526	−0.059533	0.510475	0.0552*	
H1n5	0.373839	0.715311	0.677503	0.0543*	
H2n5	0.265147	0.601891	0.658106	0.0543*	
H1n6	0.535457	0.589501	0.512718	0.0546*	
H2n6	0.536798	0.705007	0.591462	0.0546*	

### Atomic displacement parameters (Å^2^)

**Table:**

	*U*^11^	*U*^22^	*U*^33^	*U*^12^	*U*^13^	*U*^23^
Zn1	0.02747 (16)	0.02426 (15)	0.02265 (16)	0.00004 (11)	0.00160 (12)	−0.00106 (11)
Se1	0.02532 (13)	0.02427 (12)	0.01920 (13)	−0.00028 (8)	0.00161 (10)	0.00140 (11)
O1	0.0414 (12)	0.0444 (10)	0.0231 (9)	−0.0183 (9)	0.0063 (9)	−0.0069 (8)
O2	0.0396 (10)	0.0290 (9)	0.0264 (10)	−0.0047 (7)	−0.0031 (8)	−0.0027 (6)
O3	0.0268 (9)	0.0441 (10)	0.0408 (12)	0.0020 (8)	0.0042 (10)	0.0047 (10)
O4	0.0401 (11)	0.0336 (9)	0.0441 (12)	0.0049 (8)	0.0048 (10)	0.0138 (9)
S1	0.0252 (3)	0.0343 (3)	0.0323 (3)	0.0001 (2)	0.0004 (3)	0.0102 (3)
C1	0.0314 (15)	0.0266 (12)	0.0291 (14)	−0.0051 (10)	−0.0037 (11)	0.0013 (10)
N1	0.0423 (14)	0.0369 (12)	0.0412 (15)	0.0019 (10)	0.0037 (11)	0.0138 (11)
N2	0.0364 (14)	0.0400 (13)	0.0575 (18)	0.0084 (12)	0.0078 (13)	0.0189 (12)
S2	0.0354 (4)	0.0400 (4)	0.0227 (3)	0.0119 (3)	0.0055 (3)	0.0041 (2)
C2	0.0356 (14)	0.0326 (13)	0.0260 (14)	0.0060 (11)	0.0011 (11)	0.0012 (10)
N3	0.0477 (16)	0.0652 (17)	0.0276 (13)	0.0260 (14)	0.0023 (11)	−0.0063 (12)
N4	0.0435 (15)	0.0629 (17)	0.0315 (13)	0.0267 (12)	−0.0011 (11)	0.0021 (12)
S3	0.0228 (3)	0.0406 (3)	0.0279 (3)	0.0011 (2)	−0.0020 (3)	−0.0110 (3)
C3	0.0324 (13)	0.0296 (12)	0.0266 (14)	0.0035 (10)	−0.0047 (11)	−0.0045 (11)
N5	0.0384 (15)	0.0655 (17)	0.0320 (14)	−0.0047 (12)	0.0003 (12)	−0.0218 (12)
N6	0.0328 (14)	0.0620 (17)	0.0418 (16)	−0.0102 (11)	0.0010 (12)	−0.0253 (12)

### Geometric parameters (Å, °)

**Table:**

Zn1—O1	1.984 (2)	C3—N5	1.313 (4)
Zn1—S1	2.3207 (8)	C3—N6	1.321 (3)
Zn1—S2	2.3330 (8)	N1—H1n1	0.89
Zn1—S3	2.3302 (7)	N1—H2n1	0.89
Se1—O1	1.663 (2)	N2—H1n2	0.89
Se1—O2	1.6307 (18)	N2—H2n2	0.89
Se1—O3	1.6246 (19)	N3—H1n3	0.89
Se1—O4	1.621 (2)	N3—H2n3	0.89
S1—C1	1.722 (2)	N4—H1n4	0.89
C1—N1	1.326 (3)	N4—H2n4	0.89
C1—N2	1.313 (4)	N5—H1n5	0.89
S2—C2	1.724 (2)	N5—H2n5	0.89
C2—N3	1.317 (4)	N6—H1n6	0.89
C2—N4	1.306 (3)	N6—H2n6	0.89
S3—C3	1.729 (2)		
			
Se1—Zn1—S1	88.22 (2)	H1n1—N1—H2n1	120.0
Se1—Zn1—S2	115.80 (2)	C1—N2—H1n2	120.0
Se1—Zn1—S3	122.45 (2)	C1—N2—H2n2	120.0
S1—Zn1—S2	111.31 (3)	H1n2—N2—H2n2	120.0
S1—Zn1—S3	115.09 (3)	C2—N3—H1n3	120.0
S2—Zn1—S3	103.70 (3)	C2—N3—H2n3	120.0
O1—Se1—O2	105.75 (10)	H1n3—N3—H2n3	120.0
O1—Se1—O3	108.14 (11)	C2—N4—H1n4	120.0
O1—Se1—O4	108.98 (11)	C2—N4—H2n4	120.0
O2—Se1—O3	110.62 (10)	H1n4—N4—H2n4	120.0
O2—Se1—O4	110.95 (10)	C3—N5—H1n5	120.0
O3—Se1—O4	112.15 (11)	C3—N5—H2n5	120.0
S1—C1—N1	116.84 (19)	H1n5—N5—H2n5	120.0
S1—C1—N2	123.6 (2)	C3—N6—H1n6	120.0
N1—C1—N2	119.5 (2)	C3—N6—H2n6	120.0
S2—C2—N3	122.86 (19)	H1n6—N6—H2n6	120.0
S2—C2—N4	117.7 (2)	H1n1—N1—H2n1	120.0
N3—C2—N4	119.4 (2)	H1n2—N2—H2n2	120.0
S3—C3—N5	118.6 (2)	H1n3—N3—H2n3	120.0
S3—C3—N6	122.2 (2)	H1n4—N4—H2n4	120.0
N5—C3—N6	119.2 (2)	H1n5—N5—H2n5	120.0
C1—N1—H1n1	120.0	H1n6—N6—H2n6	120.0
C1—N1—H2n1	120.0		

### Table 1 Tab. 1. Hydrogen-bond geometry (Å, °). */y indicates that the pertinent hydrogen bond is also present in Zn[(SC(NH_2_)_2_]_3_(SO_4_), Krupková *et al.* (2007).

**Table:**

D-H···A	D-H	H···A	D···A	D-H···A	*
N1-H1N1···O4^i^	0.89	2.20	3.066 (3)	164	y
N1-H2N1···O3^ii^	0.89	2.38	3.072 (3)	135	y
N2-H1N2···O2^ii^	0.89	1.98	2.852 (3)	167	y
N2-H2N2···S3	0.89	2.63	3.497 (3)	166	
N3-H1N3···O3	0.89	2.17	2.988 (3)	152	y
N3-H2N3···O1^iii^	0.89	2.04	2.895 (3)	160	y
N4-H1N4···O2^iV^	0.89	2.12	2.999 (3)	168	y
N4-H2N4···S2^iii^	0.89	2.86	3.643 (3)	148	
N5-H1N5···O3^V^	0.89	2.06	2.938 (3)	170	y
N5-H2N5···O4^Vi^	0.89	2.57	3.297 (3)	139	
N6-H1N6···S1	0.89	2.73	3.577 (3)	159	
N6-H2N6···O4^v^	0.89	2.19	2.905 (3)	137	y

Symmetry codes:(i) 1/2+x,-y+1, z; (ii) x, y+1, z; (iii) 1/2+x, -y, z; (iv) 1-x, -y, 1/2+z; (v) 1-x, 1-y, 1/2+z; (vi) 1/2-x, y, 1/2+z.
